# Dose-Dependent Suppression of Human Glioblastoma Xenograft Growth by Accelerator-Based Boron Neutron Capture Therapy with Simultaneous Use of Two Boron-Containing Compounds

**DOI:** 10.3390/biology10111124

**Published:** 2021-11-02

**Authors:** Vladimir Kanygin, Ivan Razumov, Alexander Zaboronok, Evgenii Zavjalov, Aleksandr Kichigin, Olga Solovieva, Alphiya Tsygankova, Tatiana Guselnikova, Dmitrii Kasatov, Tatiana Sycheva, Bryan J. Mathis, Sergey Taskaev

**Affiliations:** 1 Laboratory of Medical and Biological Problems of BNCT, Novosibirsk State University, 1 Pirogov Str., 630090 Novosibirsk, Russia; kanigin@mail.ru (V.K.); razumov@bionet.nsc.ru (I.R.); zavjalov@bionet.nsc.ru (E.Z.); sam@211.ru (A.K.); solovieva@bionet.nsc.ru (O.S.); alphiya@yandex.ru (A.T.); guselnikova@niic.nsc.ru (T.G.); kasatovd@gmail.com (D.K.); 2 Center for Genetic Resources of Laboratory Animals, Institute of Cytology and Genetics SB RAS, 10, Acad. Lavrentieva Ave., 630090 Novosibirsk, Russia; 3 Department of Neurosurgery, Faculty of Medicine, University of Tsukuba, 1-1-1 Tennodai, Tsukuba 305-8575, Ibaraki, Japan; 4 Nikolaev Institute of Inorganic Chemistry SB RAS, 3, Acad. Lavrentieva Ave., 630090 Novosibirsk, Russia; 5 Budker Institute of Nuclear Physics, Siberian Branch of Russian Academy of Sciences, 11, Acad. Lavrentieva Ave., 630090 Novosibirsk, Russia; sychevatatyanav@gmail.com (T.S.); taskaev@inp.nsk.su (S.T.); 6 International Medical Center, University of Tsukuba Hospital, 2-1-1 Amakubo, Tsukuba 305-8576, Ibaraki, Japan; bmathis@md.tsukuba.ac.jp; 7 Laboratory of BNCT, Novosibirsk State University, 1 Pirogov Str., 630090 Novosibirsk, Russia

**Keywords:** boron neutron capture therapy, accelerator-based neutron source, boron compounds, boronophenylalanine, sodium borocaptate, glioblastoma, animal tumor model

## Abstract

**Simple Summary:**

Accelerator-based boron neutron capture therapy (BNCT) has opened up new perspectives in increasing cancer treatment efficacy, including malignant brain tumors and particularly glioblastoma. We studied dosimetry control optimization, neutron beam parameter adjustment, and two boron compound combinations (along with single and double irradiation regimens) to assess safety and increase therapy efficacy, using a U87MG xenotransplant immunodeficient mouse model. In two sets of experiments, we achieved increases in tumor-growth inhibition (to 80–83%), a neutron capture therapy ratio of 2:1 (two times higher neutron capture therapy efficacy than neutron irradiation without boron), and increases in animal life expectancy, from 9 to 107 days, by treatment parameter adjustment. These results will contribute to the development of clinical-trial protocols for accelerator-based BNCT and further innovations in this cancer treatment method.

**Abstract:**

(1) Background: Developments in accelerator-based neutron sources moved boron neutron capture therapy (BNCT) to the next phase, where new neutron radiation parameters had to be studied for the treatment of cancers, including brain tumors. We aimed to further improve accelerator-BNCT efficacy by optimizing dosimetry control, beam parameters, and combinations of boronophenylalanine (BPA) and sodium borocaptate (BSH) administration in U87MG xenograft-bearing immunodeficient mice with two different tumor locations. (2) Methods: The study included two sets of experiments. In Experiment #1, BPA only and single or double irradiation in higher doses were used, while, in Experiment #2, BPA and BSH combinations and single or double irradiation with dosage adjustment were analyzed. Mice without treatment or irradiation after BPA or BPA+BSH injection were used as controls. (3) Results: Irradiation parameter adjustment and BPA and BSH combination led to 80–83% tumor-growth inhibition index scores, irradiation:BNCT ratios of 1:2, and increases in animal life expectancy from 9 to 107 days. (4) Conclusions: Adjustments in dosimetry control, calculation of irradiation doses, and combined use of two ^10^B compounds allowed for BNCT optimization that will be useful in the development of clinical-trial protocols for accelerator-based BNCT.

## 1. Introduction

Human glioblastomas are the most common malignancies with low median patient survival rates. These tumors are aggressive, prone to recurrence, radioresistant, and poorly treated with standard protocols [1]. In recent decades, the use of boron neutron capture therapy (BNCT) in patients with such tumors has demonstrated encouraging results [2,3,4,5]. BNCT is a binary radiotherapy method based on the selective interaction of a stable isotope (^10^B) and a neutron, resulting in a nuclear reaction delivering energy in a small area corresponding to the radius of mammalian cells [6]. Sufficient delivery of the ^10^B isotope to tumor cells contributes to their targeted destruction and largely determines the success of BNCT [7]. Over the past decades, clinical studies on glioma BNCT have been conducted in Finland at the FIR1 reactor, in Japan at the JRR-4 and KUR reactors, and in Taiwan at the THOR reactor [8]. These initial studies showed encouraging results compared to conventional radiotherapy treatment and concomitant administration of temozolomide [9]. However, most nuclear reactors used for BNCT were closed due to safety concerns, or political and economic reasons, rather than any clinical results.

To further develop BNCT, leading research groups focused on development of a safe source of epithermal neutrons, along with a search for effective ^10^B targeted delivery compounds. Thus, several types of charged particle (proton) accelerators capable of neutron production and suitable for installation in a hospital or a specified BNCT center have been developed [10,11,12,13,14,15]. Based on these developments, several clinical BNCT centers have been established. The first accelerator-based BNCT system in Europe was installed at Helsinki University Hospital (HUH) in Finland in 2019, with an accelerator constructed by Neutron Therapeutics, Inc. (Danvers, MA, USA); meanwhile, in Japan, Sumitomo Heavy Industries, Ltd. (Tokyo, Japan) constructed a neutron source for BNCT. Based on a cyclotron, Sumitomo has successfully completed initial clinical testing of their facility (Phase II) and started patient treatment under the Japanese national health insurance system in 2020, using the first registered boron drug (Steboronine^®^, produced by Stella Pharma, Inc., Tokyo, Japan) [12,16,17]. At the Budker Institute of Nuclear Physics (BINP, Novosibirsk, Russian Federation), an original accelerator-based neutron source has been installed [18,19,20,21] that meets BNCT requirements [1,6]. This accelerator is a prototype of the accelerator by TEA Life Sciences (Foothill Ranch, Lake Forest, CA, USA) that is being constructed for clinical use [14]. The beam parameters achieved to date allow preclinical studies to evaluate the effectiveness of BNCT in vitro and in vivo, while methods for testing stable epithermal neutron generation and dosimetry at this accelerator are being developed to achieve the necessary conditions for its further use in clinical trials [22,23,24,25,26].

The efficacy of BNCT is also directly related to ^10^B compounds, as they should be non-toxic, accumulate predominantly in the tumor with a ^10^B tumor/normal tissue difference >3:1 or in a ^10^B concentration of 20–50 µg/g tumor, be relatively quickly eliminated from blood and normal tissues, and remain in the tumor for the few hours required for the neutron irradiation process [7,27]. Two low-molecular-weight ^10^B-containing compounds have been used in clinical trials, boronophenylalanine (BPA, C_8_H_7_NH_2_COOH-B(HO)_2_) and sodium borocaptate (BSH, Na_2_B_12_H_11_SH), but innovation in new compounds is a very complex task requiring time, effort, and substantial financial investment. Until recently, none of the newly reported compounds has been cleared for clinical application [28,29,30,31,32].

Though BPA and BSH are not perfect regarding the ideal requirements for BNCT, several groups worldwide have confirmed their efficacy in glioma, head and neck cancer, melanoma, and metastatic colon cancer treatment [2,3,4,5,6,33,34,35,36,37,38]. Combined administration of BPA and BSH previously proved to be more effective in increasing tumor ^10^B concentration during in vivo experiments [39,40], while specific protocols to maximize the efficacy of combinations of these two compounds have been reported in reactor-based clinical trials [4,41,42]. Therefore, in our study, we also proposed BPA and BSH co-administration to improve ^10^B tumor accumulation.

Along with the proper selection and combination of ^10^B compounds, proper dosimetry control and determination of the calculated radiation parameters aimed at reducing the radiation burden on healthy tissues are important aspects of successful therapy. Optimized dosimetry control opens prospects for enhancing the effectiveness of BNCT [21], as it was found that irradiation from two or more points makes it possible to achieve the required level of tumor irradiation while minimizing the impact on surrounding tissues [43].

Thus, the aim of this study was two-fold. The first was to achieve higher BNCT efficacy by increasing total ^10^B accumulation in tumor tissue via the sequential injection of two ^10^B-containing drugs (BPA and BSH), while the second relied on the comparison of two different irradiation regimens to analyze and improve the safety and efficiency of accelerator-based BNCT. In this study, we intentionally used subcutaneous tumor models in order to distance the irradiation zone from vital organs and obtain animal life expectancies sufficient to fully assess the effectiveness of BNCT.

Since the accelerator is unique and the use of variants for clinical applications has not been reported, we are the first to test the proposed protocols and dosimetry approaches in animal experiments at an accelerator neutron source.

## 2. Materials and Methods

The study included two sets of experiments, designated hereafter as Experiment #1 and Experiment #2.

### 2.1. Cell Cultures

Human glioblastoma U87 MG cells (ATCC^®^ HTB-14™) were obtained from the collection of the SPF Vivarium Collective Use Center of the Institute of Cytology and Genetics SB RAS (ICG SB RAS) (Novosibirsk, Russia). The cells were cultured in DMEM/F12 (1:1) medium (BioloT Ltd., St. Petersburg, Russia) with the addition of 10% Gibco fetal bovine serum (FBS, Thermo Fisher Scientific, Waltham, MA, USA) at 37 °C in a 5% CO_2_ atmosphere.

### 2.2. Preparation of Tumor Xenografts

Male SCID mice with SPF health status at 5–7 weeks of age were subcutaneously inoculated with 100 μL of a human U87MG glioblastoma cell suspension. In Experiment #1 (*n* = 32), 6 million U87 MG cells were injected in the scapula region of each mouse, and, in Experiment #2 (*n* = 40), 3 million cells were subcutaneously injected into the thigh to separate the location of the xenograft irradiation area from vital organs. Then, the growth of tumor nodules was monitored, and their linear dimensions were measured by using an electronic caliper (Rosinstrument, Co. Ltd., Novosibirsk, Russia).

The xenograft volume (V) was calculated according to the following formula:

V = (a × b^2^) × 0.52, (1)

where a is the length, and b is the width of the tumor [44].

Two weeks after cell inoculation, the animals were divided into groups and irradiated at the accelerator-based neutron source. Both experiments contained 6 groups each: “Control” (no treatment), “Control+BCD” (BCD was the injected boron-containing drug or drugs: BPA in Experiment #1 or BPA+BSH in Experiment #2), “Irradiation-1” (single irradiation without BCD), “Irradiation-2” (double irradiation without BCD), “BNCT-1” (BNCT with single irradiation after BCD injection), and “BNCT-2” (BNCT with double irradiation after BCD injection).

After irradiation, the volumes of xenografts and body weights of the animals were measured every 2 to 3 days, and the tumor growth inhibition (TGI) index was calculated according to the following formula: TGI (%) = ((Vcont − Vexp)/Vcont) × 100, where Vcont is the average tumor volume in the Control group, and Vexp is the average tumor volume in the experimental group [44].

Animal experiments were approved by the Institutional Review Board (Institute of Cytology and Genetics SB RAS; see the Institutional Review Board Statement), and all manipulations were performed respecting the principles of humane treatment of animals (European Community Directive 86/609/EEC). Planned euthanasia was performed by CO_2_ overdose.

### 2.3. Boron Compounds

BPA (Interpharma Praha A.s., Praha, Czech Republic) and BSH (Katchem spol. sr. o., Praha, Czech Republic) enriched with isotope ^10^B (99%) were used as boron compounds. The BPA solution was prepared with the addition of fructose (Sigma-Aldrich, Inc., St. Louis, MO, USA) according to a previously described protocol [6]. BSH was dissolved in 0.9% NaCl (Renewal, Ltd., Novosibirsk, Russia) according to the manufacturer’s instructions.

In Experiment #1, only BPA solution at a dose of 700 mg/kg was used. In Experiment #2, in order to increase the boron dose, two solutions were co-administered as two injections: BPA 700 mg/kg and BSH 100 mg/kg. The compounds were administered 0.5 h before irradiation intraperitoneally to animals from 3 groups: Control+BCD, BNCT-1, and BNCT-2.

### 2.4. Irradiation Experiments

The animals were irradiated at the source of epithermal neutrons, which included an electrostatic tandem accelerator with vacuum insulation [18,20], a lithium neutron-generating target (reaction ^7^Li(p,n)^7^Be) [21,45], and a neutron-beam shaping assembly [21,46]. The characteristics of the neutron beam and its contamination by fast neutrons and photons have been described previously [47,48]. Spatial distribution of the beam components was studied parallel to the current research, using a water phantom explicitly set up to provide experimental rather than computational data [49].

Animal positioning and irradiation settings were arranged as described in our previous work [26]. Briefly, to irradiate mice, a special plexiglass moderator was positioned between the target and the mice to ensure maximum thermal neutron density in the tumor area. The mice were fixed in plastic restraints and placed radially in a 7.5% lithium polyethylene container, with the right thigh secured to the center of the container. The lid of the container covered their bodies, exposing only the leg with the tumor, to reduce radiation exposure to non-target areas. The container was located at a distance of 0.5 cm from the 7.4 cm plexiglass moderator attached to the target. The animals were irradiated by a neutron beam with the following accelerator operation parameters: proton energies of 2.1 (Experiment #1) and 2.05 (Experiment #2) MeV, proton current integral of 5.5 mAh in Experiment #1 in the single and in each of both fractions in double irradiation, and 3.4 mAh in the single irradiation in Experiment #2, and 2.3 mAh in each of both fractions in double irradiation, respectively. Animals from the Irradiation-1 and BNCT-1 groups were irradiated once while animals from the Irradiation-2 and BNCT-2 groups were irradiated 2 times, each time on different days.

### 2.5. Calculation of Boron-Related Absorbed Dose

To calculate the boron dose, the concentrations of boron in the tumor, blood, muscle tissue, brains, kidneys, livers, and spleens of mice were determined. Before each experiment, a separate group of animals with xenografts was intraperitoneally injected with the same drugs that were then used in the experiment. After 1.5 and 3 h (Experiment #1) and 1 and 2 h (Experiment #2), the animals were sacrificed, and tissue samples were collected; the samples were stored frozen. Boron concentration was determined at the Institute of Inorganic Chemistry (Novosibirsk, Russia), using a high-resolution iCAP-6500 spectrometer (Thermo Fisher Scientific, Inc., Waltham, MA, USA) according to specially developed methods of sample preparation and quantitative chemical analysis [50].

### 2.6. Exposure Parameters

In BNCT, the total absorbed radiation dose consists of four main components: the gamma-ray dose (from gamma rays produced in neutron interactions and from interaction of protons with the lithium target), the dose emitted as a result of neutron capture by boron-10 (^10^B(n,α)^7^Li, the so-called “boron dose”), the dose from fast neutrons (arising mainly from the elastic neutron scattering reaction on hydrogen), and the dose from thermal neutrons, the main source of which is the ^14^N(n,p)^14^C reaction [6].

Table 1 shows the total absorbed dose in the tumor (last column) and its component calculated per mA × hour: 5.5 for the first experiment and 3.4 and 2.3 for the single and double irradiation of the 2nd experiment.

When calculating the absorbed dose, these components were taken into account regarding RBE (relative biological effectiveness) and CBE (compound biological effectiveness) coefficients. RBE for neutrons is assumed to be 3.2, and gamma rays are 1, while the CBE coefficients for healthy tissue and tumors are assumed to be 1.3 and 3.8 [51]. The boron concentration was also taken into account when calculating the boron dose. Radiation doses were calculated by using the NMC code for photon and neutron transport by the Monte Carlo method [52].

### 2.7. Statistical Analysis

The data were statistically processed by using STATISTICA-10 software (StatSoft Inc., Tulsa, OK, USA, 2011). All data are presented as mean values with confidence intervals taken into account. The unpaired Student’s *t*-test was used to compare normally distributed quantitative data. The Mann–Whitney test was used to compare non-normally distributed quantitative data. Data on overall animal survival are graphically presented as Kaplan–Meier curves. The log-rank test was used to compare survival curves. Differences were considered statistically significant at *p* < 0.05.

## 3. Results

### 3.1. Compound Biodistribution

#### 3.1.1. Experiment #1

**Figure 1:**
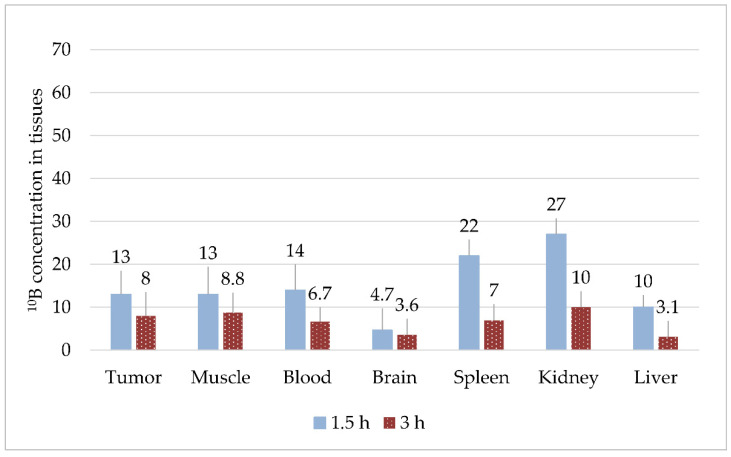
Boron content in the organs of mice 1.5 and 3 h after BPA injection, µg/g.

With intraperitoneal injection of BPA solution at a dose of 700 mg/kg, the boron concentrations for all organs were significantly higher at 1.5 h versus 3 h (*p* < 0.05, Figure 1), with the highest values observed in the spleen and kidneys. The relative tumor/blood and tumor/brain ratios in Experiment #1 for the 1.5 h timepoint were 0.9 and 2.8, respectively.

#### 3.1.2. Experiment #2

**Figure 2:**
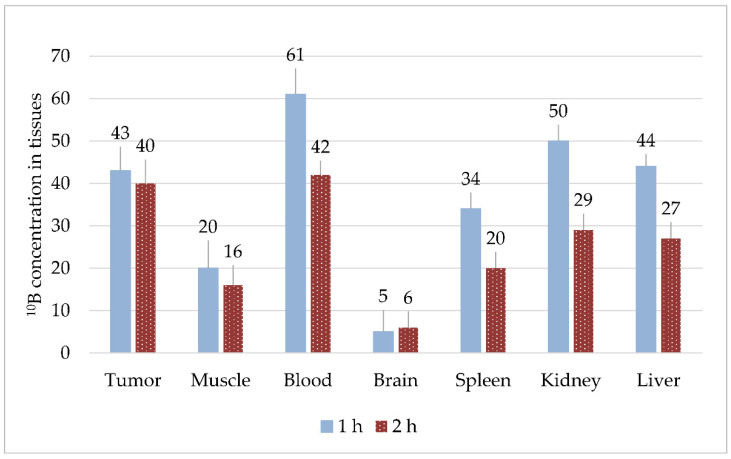
Boron content in the organs of mice 1 and 2 h after co-administration of BPA and BSH solutions, µg/g.

With intraperitoneal injection of two solutions (BPA at a dose of 700 mg/kg and BSH at a dose of 100 mg/kg), the distribution of boron in the organs changed. High boron concentrations (>30 µg/g) were recorded in tumors, blood, spleens, kidneys, and livers 1 h after co-administration (Figure 2). We observed that, in blood, spleen, kidney, and liver, boron concentrations after 2 h were 1.4–1.7 times lower than after 1 h (*p* < 0.05), whereas, for the tumor, muscle, and brain, the results were not statistically significant. The highest concentrations were observed in the blood and kidneys, but relative values of tumor/blood and tumor/brain ratios in Experiment #2 also changed from Experiment #1, with ratios of 0.7 and 9.2, respectively.

### 3.2. Irradiation Experiments

#### 3.2.1. Experiment #1

**Figure 3:**
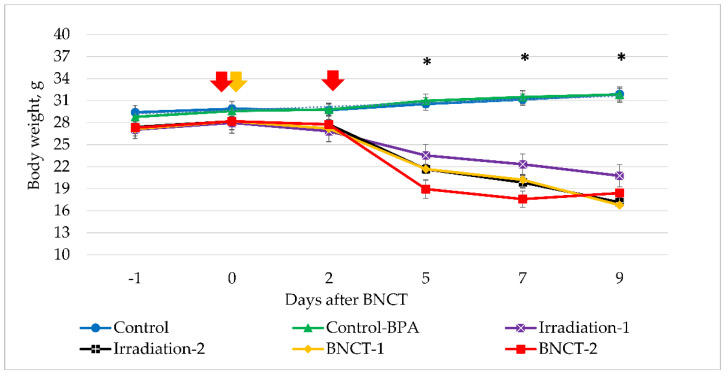
Average body weight of Control versus experimental animals in all groups (mean ± SE). Arrows indicate days when neutron irradiation took place. * Statistically significant differences (*p* < 0.05) for Irradiation-1, Irradiation-2, BNCT-1, and BNCT-2 groups compared to the Control.

As shown in Table 1, the irradiation doses (17.6 and 35.2 Gy-Eq) in this experiment were intentionally selected to be high in order to determine the maximum permissible irradiation dose. As a result, clinical examination of the animals on day 5 after exposure revealed significant deterioration in animal condition. In the Irradiation-2 group, hunched bodies and diarrhea were noted in 100% of animals, while, in the BNCT-1 group, 30% of animals exhibited hunched bodies and 60% had diarrhea. In the BNCT-2 group, hunched bodies were observed in 70% of the mice and diarrhea in 15%. At the same time, a significant decrease in average body weight was observed in all groups of animals that received irradiation (Figure 3).

In addition, in all irradiated groups in Experiment #1, animal death was observed on days 5–9. As a result, on the 10th day after irradiation, there were no surviving mice in the Irradiation-2, BNCT-1, or BNCT-2 groups, and this seems to correlate with the highest radiation dose received from the accelerator (Table 1). Of the two animals that survived in the Irradiation-1 group, only one survived till the endpoint of the experiment 23 days after irradiation. Based on these observations, the beam parameters for single irradiation can be characterized as LD80, while the parameters for double irradiation were deemed LD100.

Non-irradiated Control and Control+BPA groups showed no reductions in body weight during the entire observation period, and no clinical signs of toxicity of the injected compounds were observed in the animals of the Control+BPA group; apparently, a single intraperitoneal injection of BPA at a dose of 700 mg/kg had no effect on the animals. All mice from the non-irradiated Control groups were euthanized and autopsied according to bioethical standards (maximum allowable volume of xenografts) on the 23rd day after exposure.

#### 3.2.2. Experiment #2

**Figure 4:**
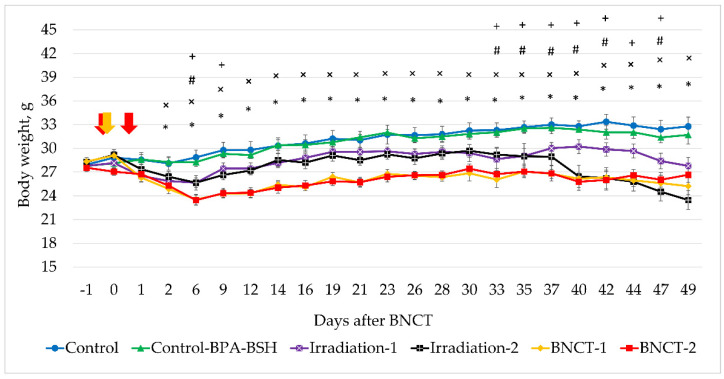
Average body weight of Control versus experimental animals in all groups (mean ± SE). Arrows indicate days when neutron irradiation took place. *p* < 0.05: *—significant difference between BNCT-1 and Control; #—between Irradiation-1 and Control; ×—between BNCT-2 and Control; +—between Irradiation-2 and Control.

As shown in Table 1, the radiation doses in the BNCT-1 and BNCT-2 groups in this experiment were reduced two and three times and amounted to 8.1 and 11.0 Gy-Eq, respectively. In addition, the irradiation area was moved away from the scapular region to the hind limbs, far from the vital organs. However, despite the measures taken, by day 6, all the exposed animals had lost 10–15% of their initial body weight (Figure 4), with no other clinical signs of health deterioration. Then, from days 7 to 40, the body weight of the animals in these groups stabilized, but, after 40 days, the Irradiation-2 group experienced another weight decrease. After 44 days, all irradiated mice exhibited signs of hunched bodies.

**Figure 5:**
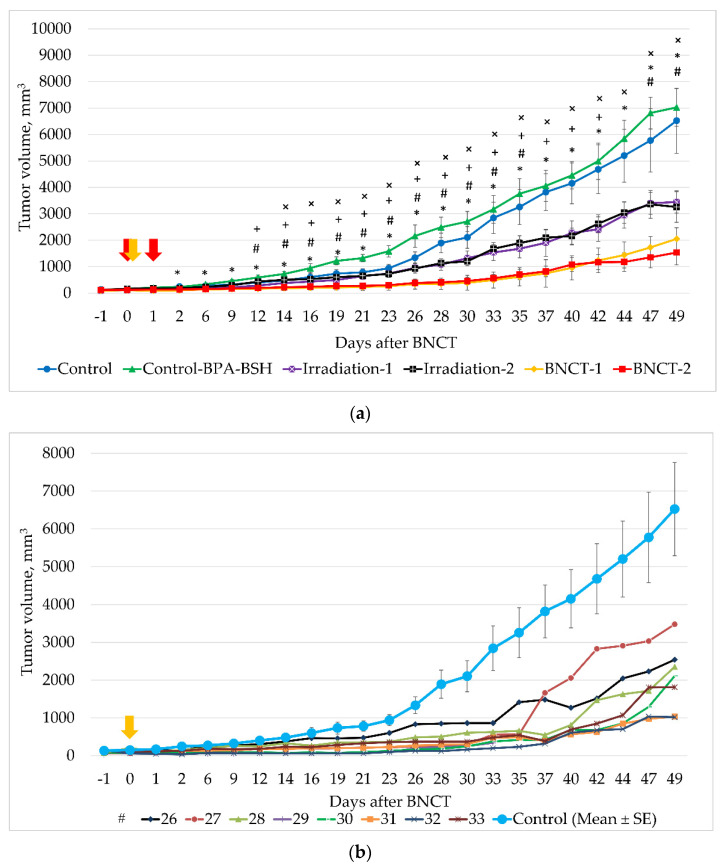

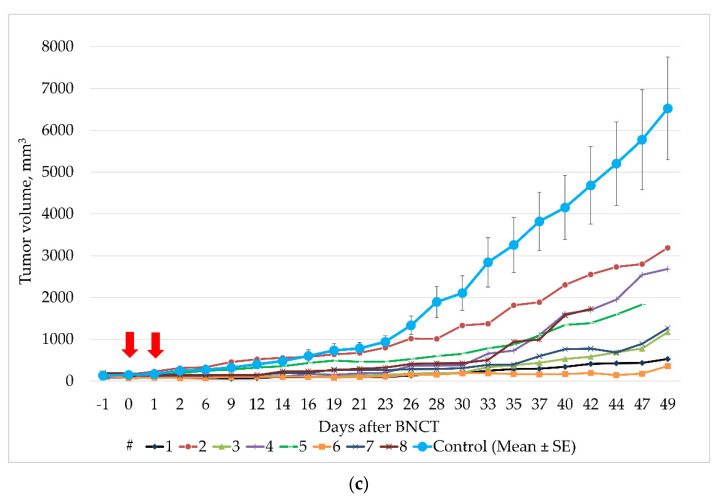
Comparative evaluation of the effects of BNCT and irradiation on the growth of U87MG xenografts in SCID mice. (**a**) Determination of xenograft growth in animal groups, mean ± SE; (**b**) individual growth of U87MG xenografts in mice after single BNCT; and (**c**) individual growth of U87MG xenografts in mice after double BNCT. The arrows indicate the days on which neutron irradiation took place. *p* < 0.05, Z = 2.02: *—significant differences between the BNCT-1 and Control; #—between BNCT-1 and Irradiation-1; ×—between BNCT-2 and Control; +—between BNCT-2 and Irradiation-2.

As shown in Figure 5a, the average volume of xenografts after single BNCT, starting from the second day, significantly differed from the values of the Control group (2.4 times lower [*p* = 0.023]) and, from 12 days, significantly differed from the values in the Irradiation-1 group (*p* = 0.033). Dual BNCT also proved to be effective in suppressing tumor growth from the first day; however, first-day differences in the Control and Irradiation-2 groups were significant only from 14 and 12 days, respectively (*p* = 0.048 and 0.033, respectively). The average tumor volume in the BNCT-2 group was 3.5 and 2.8 times lower than in the Control and Irradiation-2 groups, respectively.

The maximum TGI values, as determined on the 33rd day after irradiation, were 83% for the BNCT-1, 80% for the BNCT-2, 46% for the Irradiation-1, and 41% for the Irradiation-2 groups. At the Control group euthanasia endpoint (day 49), the TGI values were 69% for the BNCT-1, 76% for the BNCT-2, 47% for the Irradiation-1, and 50% for the Irradiation-2 groups.

The individual growth of xenografts in animals from BNCT-1 and BNCT-2 groups is shown in Figure 5b,c. In the BNCT-1 group, two out of eight animals on day 49 had relatively small xenograft volumes (1033 and 1021 mm^3^ (#31 and #32)). Comparing these values with the average volumes of xenografts of the Control group on day 49, we obtained TGI values equal to 84%.

In the BNCT-2 group, the minimal tumor volumes on the 49th day in two animals were 530 and 360 mm^3^ (#1 and #6), which was 12 and 18 times less than the average values in the Control group, and corresponded to TGIs of 92% and 94%, respectively. On day 69 after irradiation, the tumor volumes in these mice were 764 and 170 mm^3^, corresponding to TGIs of 88% and 97%. Thus, 10 weeks after double BNCT, the tumor volume remained almost the same as at the start of the experiment in one out of eight mice.

Animals from the Control and Control+BCD (BPA+BSH) groups had a normal body weight but showed more intensive growth of xenografts than in experimental groups during the whole period of observation. In this regard, on day 49, these groups were withdrawn from the experiment according to bioethical standards (maximum allowable volume of xenografts).

On day 49, the number of surviving animals for the BNCT-1 group was seven out of eight (87%), BNCT-2 was six out of eight (75%), Irradiation-1 was six out of seven (86%), and Irradiation-2 was three out of seven (43%). These groups continued to be monitored. The Irradiation-2 group was completely withdrawn from the experiment on day 54, and mice from the remaining groups died on different days between days 49 and 107.

**Figure 6:**
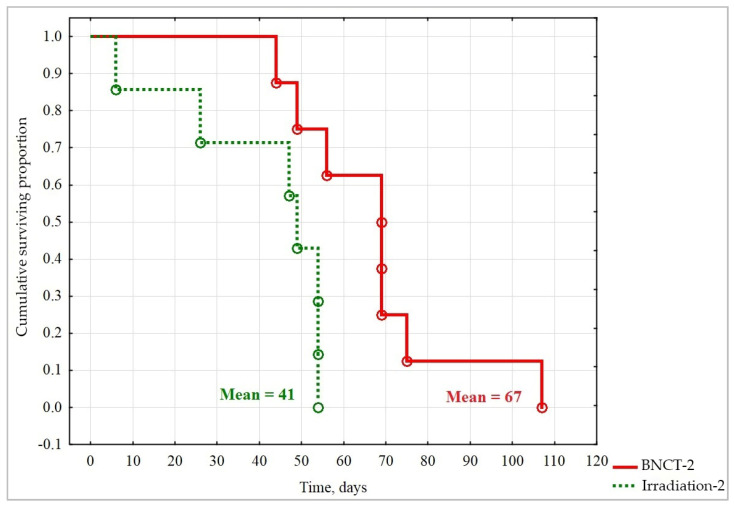
Kaplan–Meier curves of overall survival of animals in the two groups; ○—animal death. Differences in overall survival are statistically significant, log-rank test, *p* = 0.0275.

To compare the overall survival of animals from different groups, Kaplan–Meier survival curves were plotted and compared, using the log-rank criterion. Statistically significant differences were found only between the Irradiation-2 and BNCT-2 groups (Figure 6). Thus, double BNCT with the abovementioned parameters (Table 1) increased the lifespan of immunodeficient SCID mice by 26 days, as compared to double irradiation.

## 4. Discussion

Due to the global transition of BNCT research to the use of accelerator-based neutron sources, research groups in Japan, Finland, Taiwan, and other countries that previously conducted experiments at reactors are adapting to these new conditions. In order to develop new protocols to establish clinical-scale trials, the optimization of both compound and neutron beams are especially important in light of this shift away from reactor-based sources. We believe that, based on the results of previous trials at nuclear reactors and accelerator-based neutron sources by other research groups [2,3,4,5,43,53,54], as well as our own data [19,21,22,23,24,26,50,55], biodistribution of boron-containing compounds with regard to accelerator-based neutron sources is the current and well-reported future of BNCT [26,43]. It was thus necessary to continue the selection of optimal conditions for such experiments.

With regard to the neutron source switch, determining LD100% in the setting of BNCT for immunodeficient mice is a crucial first step in establishing effective in vivo modeling. As in previous reports [23,26,50], this study demonstrates the presence of high-energy ionizing components in the beam. Additionally, despite the fact that BNCT theoretically asserts that irradiation of healthy tissues without boron is considered safe, in practice, we encountered enhanced deterioration of animal condition based on tumor localization, as the scapula region used in the present study differed from the hind paw on the outer side chosen in a previous study [50].

BPA and BSH differ in their chemical structure, properties, and bioaccumulation in tumor tissue [6]. BPA penetrates tumor cells through an amino acid metabolism pathway, with the help of L-amino acid transporters 1 and 2 [56,57], thus limiting higher boron accumulation to intensively dividing tumor cells with high metabolism. Therefore, BPA has been one of the best candidates for malignant glioma treatment, along with its property of passing through the blood–brain barrier (BBB). However, due to this nature, BPA might not accumulate efficiently in resting tumor cells, thus limiting its effective use [39,58]. BSH, on the other hand, penetrates tumor tissue through fenestrated capillaries by adopting the enhanced permeability and retention (EPR) effect [59,60] and thus can provide non-selective homogenous ^10^B distribution in tumor tissue and tumor vessels, even while unable to pass the BBB. The issue of overcoming the BBB by using various additional chemical agents was addressed in several fundamental studies [60,61,62,63,64,65]; however, BPA and BSH co-administration tends to be more effective in reaching higher ^10^B tumor levels, as these drugs can synergistically reinforce individual weaknesses.

Separate and combined use of BPA and BSH has been studied in murine sarcoma. Wittig et al. (2008) demonstrated effective boron uptake by tumor cells and showed ^10^B transport into the tumor-cell cytoplasm and the nucleus with homogeneous (by BSH) and heterogeneous (by BPA) ^10^B distribution [66]. In another study, one-week-old mice with implanted U87 malignant glioma and S3 human sarcoma were used [67], revealing that BPA and BSH accumulate in tumor tissues at optimal concentrations recommended for BNCT, according to the EORTC protocol [68]. Given the different pharmacokinetics and pharmacodynamics of these two compounds, different injection regimens prior and during irradiation to maximize timely tumor ^10^B concentration have been utilized, and this was reflected in the clinical protocols. Thus, a combination of these agents has been successfully used in glioma therapy and clinical BNCT research protocols at nuclear reactors, with adjusted infusion or injection time before and/or during neutron irradiation [4,41,42].

In our previous in vivo study, we reported that absolute boron concentrations in the tumor could reach 28 µg/g in some mice when only BPA was administered intraperitoneally [50]. In the current study, we observed increased ^10^B concentrations in the target tissues by combining BPA and BSH administration, which is consistent with the literature data on other models [39,40]. Thus, we support the findings that BPA and BSH combination is effective in increasing the boron dose and the absolute boron content in the tumor may reach, on average, three-times higher levels than single drug administration.

It is also worth mentioning the limitations of this study. The U87MG tumor model in SCID mice is only one of multiple different possibilities, as the method is often utilized for treatment studies of other tumors, such as melanomas or head and neck cancers. However, this animal model has been previously used in a large number of preclinical experiments worldwide. In our study, we specifically used subcutaneous models of these tumors to assess treatment safety from the standpoint of tumor location and distance of irradiation from vital organs. In terms of the tumor model closest to clinical conditions in humans, an intracerebral model would be the most appropriate. However, subcutaneous xenografts were used in the current study in order to move the irradiation area further from vital organs, as using the orthotopic U87 model previously showed that animal irradiation with the parameters that we used (similar to those in Experiment #1 in this study) does not permit a life expectancy sufficient to fully assess the effectiveness of BNCT.

We were unable to fully compare the effects of treatment in the BNCT-1 and BNCT-2 groups. This was due to the fact that high doses of radiation were used in the BNCT-1 experiment, resulting in a high lethality: animals from the BNCT-1 group (BPA) lived only 9 days after exposure. We considered this period insufficient for a meaningful comparison with the BNCT-2 group (BPA/BSH), in which animals lived on average 67 days after irradiation. Additionally, drug-injection parameters were adjusted during the experiments to better comply with the irradiation timing, and this complicated direct boron-accumulation comparisons between experimental groups. However, this allowed us to reduce the number of animals needed for experiments in accordance with ethical principles, thus avoiding unnecessary irradiation with less effective parameters.

During irradiation, neutron-beam contamination by fast neutrons and photons could have played a role in the study results. According to calculations presented in Table 1, the sums of the additional beam component doses were comparable to the boron-related absorbed doses, when the latter should (ideally) be significantly higher. This could be related to the irradiation settings with the plexiglass phantom placed between the neutron producing target and the animal box, as the moderator plays a significant role in neutron-beam optimization. Fast neutrons are an extension of the proton beam, and, with regard to “harmful” doses, fast neutrons contribute the most, but their function decreases rapidly as distance increases from the center of the target. Moreover, with the increase in proton energy, the fast neutron yield increases significantly, and its dose component grows even faster, with the angular distribution becoming more uniform, causing additional, unwanted irradiation effects. Therefore, animal leg placement close to the center of the target, where the fast neutron component is the largest, also played a role in additional irradiation unrelated to BNCT. As such, Experiment #1 resulted in higher radiation exposure due to the higher proton energy, and, in both experiments, additional irradiation doses to murine bodies were relatively high, even with shielding against neutrons. Nevertheless, the presented doses were only calculated parameters and could differ from the actual conditions of an experiment.

We concluded that, before irradiating the mice, beam verification, with its component spatial distribution and volumetric dose-distribution analysis, is important. Therefore, in a recent study, spatial distribution of the beam components from this accelerator was experimentally analyzed by using a water phantom to provide actual beam-component distribution in comparison to the calculated parameters [49]. Those results will be used for dose calculation in further radiobiological experiments at the Budker Institute of Nuclear Physics accelerator, and these methods can be applied to other accelerator-based neutron sources.

Our use of BPA and BSH is not novel in itself, but, combined with our unique application and delineation of the parameters of accelerator-based neutrons, our results pave the way for clinical trials that will most likely use already-approved versus novel compounds. In our study, we obtained similar tumor/blood ratios in all experiments, suggesting the possibility of estimating accumulated ^10^B concentration in tumor tissue by boron content in the blood. This was previously suggested in clinical trials [6,68], though this could also indicate the inconsistency of the used compounds and their inability to create tumor concentrations greater than in blood in our animal tumor model.

The boron doses used in both experimental groups should have been unified. However, we used a BPA dose of 700 mg/kg as the maximum tolerable dose, based on a previous in vivo toxicity assessment of different BPA and BSH doses [26], for our experiments. The BSH dose of 100 mg/mL was previously used in reactor-based clinical trials [69] and also in our previously reported experimental protocol [26]. Thus, even if the doses were not unified, we did establish up to a three-fold increase in tumor boron concentration by using mixed doses. As the aim of this study was to increase treatment efficacy rather than simply compare different combinations of boron drugs, we used the maximum tolerable, ethical, and reported BPA and BSH doses.

Another limitation might be related to the need for additional animal experiments and irradiation/drug combinations; however, due to the limited availability of accelerator machine time, all possible combinations of experimental conditions could not be covered and may thus be successfully implemented in future studies.

## 5. Conclusions

In this study, we analyzed and compared single and mixed boron compound injections for more effective tumor boron accumulation and several irradiation regimens of the accelerator-based neutron source designed for BNCT, using a U87MG tumor model in immunodeficient SCID mice. We showed that, with mixed compound use and neutron irradiation divided into two fractions (combined with lower proton energy), higher safety and efficacy of BNCT was achieved, resulting in significant extensions to experimental lifespan and reductions in tumor growth. The analysis of the spatial distribution of the beam components and their volumetric doses is thus necessary before radiobiological experiments. These findings will be crucial in the development of clinical trial protocols for accelerator-based BNCT and will generally contribute to the development of BNCT as an effective method to treat aggressive and chemotherapy-resistant cancers.

## Figures and Tables

**Table 1:** Irradiation parameters in the experiments.

Experimental Groups	Thermal Neutrons, Gy-Eq	Fast Neutrons, Gy-Eq	Boron Dose from Neutron Capture by Boron, Gy-Eq	Gamma-Ray Dose, Gy-Eq	Average Tumor Boron Concentration, µg/g	Total Absorbed Dose, Sv
Experiment #1 (proton energy 2.1 MeV)						
BNCT 1	1.7	6.2	15.3	9.7	15	32.6
Irradiation 1	1.7	6.2	0	9.7	0	17.6
BNCT 2	1.7/3.4	6.2/12.2	15.3/30.6	9.7/19.4	15	65.8
Irradiation 2	1.7/3.4	6.2/12.2	0	9.7/19.4	0	35.2
Experiment #2 (proton energy 2.05 MeV)						
BNCT 1	0.9	2.2	6.6	5	42	21.3
Irradiation 1	0.9	2.2	0	5	0	8.1
BNCT 2	0.6/1.2	1.48/2.96	4.5/9.0	3.4/6.8	42	28.8
Irradiation 2	0.6/1.2	1.48/2.96	0	3.4/6.8	0	11.0

## Data Availability

The data presented in this study are available upon request from the corresponding author.
